# Levodopa-induced dyskinesia is associated with worse gait and balance in Parkinson’s disease

**DOI:** 10.1007/s00702-026-03217-w

**Published:** 2026-06-30

**Authors:** Gabriele Imbalzano, Federico D’Agata, Claudia Ledda, Carlo Alberto Artusi, Marco Laudadio, Alberto Romagnolo, Mario Giorgio Rizzone, Leonardo Lopiano, Maurizio Zibetti, Marco Bozzali

**Affiliations:** 1 Department of Neuroscience “Rita Levi Montalcini”, University of Turin, Via Cherasco 15, 10126 Turin, Italy; 2 SC Neurologia 2U, AOU Città della Salute e della Scienza, Turin, Italy; 3 Department of Neuroscience Biomedicine Movement, University of Verona, P. le L.A. Scuro 10, Verona, Italy

**Keywords:** Parkinson’s disease, Dyskinesia, Gait, Resting-state fMRI

## Abstract

Levodopa-induced dyskinesia (LID) is a major source of disability in advanced Parkinson’s disease (PD), whose impact on gait and balance across medication states still remains unclear. We aimed to compare gait and balance performance between comparable dyskinetic (Dysk-matched) and non-dyskinetic (No-Dysk) PD patients in OFF and ON state, and to explore neural correlates using structural and resting-state functional MRI (rs-fMRI). PD patients were consecutively enrolled among candidates for device-aided therapies. Gait and balance were assessed using validated wearable inertial sensors during a 2-minute walk test, a 3-meters Timed-Up-and-Go (TUG), and a sway test, providing spatiotemporal gait parameters, variability, asymmetry, and postural sway metrics. Groups were compared using propensity score matching (PSM) for age, sex, disease duration, motor severity and axial disability. A subgroup underwent exploratory investigation with structural and rs-fMRI collected in ON state. After PSM (22 pairs), Dysk-matched group showed slower gait speed (*p = 0.007*), shorter stride length (*p = 0.006*), longer double support (*p = 0.047*), and prolonged TUG (*p = 0.049*) in the OFF state. Dysk-matched patients showed increased sway from OFF to ON (*p = 0.030*). Neuroimaging (10 Dysk-matched, 9 No-Dysk) revealed increased functional connectivity in sensorimotor regions and reduced SMA connectivity within a cerebellar network in Dysk-matched patients, without structural difference. Overall, the association of LID with impaired gait and postural control even in the OFF state is consistent with the hypothesis of broader motor network dysfunction beyond overt involuntary movements.

**Supplementary Information:**

The online version contains supplementary material available at https://doi.org/10.1007/s00702-026-03217-w.

## Background

Levodopa-induced dyskinesia (LID) is a common motor complication in Parkinson’s disease (PD), typically emerging after 4–6 years of levodopa therapy, with a prevalence rising from 30% to 40% at early disease stages to nearly 80% at advanced stages (Tran et al. 2018; Turcano et al. 2018; Armstrong and Okun 2020).

Clinically, LID most often presents with choreiform movements at peak levodopa concentrations (“peak-dose dyskinesia”), but it can also manifest as diphasic dyskinesia or off-period dystonia occurring during low dopaminergic states (Marsden et al. 1982).

Beyond its motor manifestations, LID has important consequences for postural control and fall risk (Curtze et al. 2015; Rascol et al. 2015). While levodopa can improve gait speed and step length, it paradoxically worsens balance in patients with LID, resulting in increased sway during both quiet stance and dynamic tasks (Curtze et al. 2015; Revilla et al. 2013; Bonnet et al. 2017), with consequent instability and a higher risk of falls (Curtze et al. 2015; Cenci et al. 2020). Most studies have focused on ON-state performance. However, experimental evidence indicates that dyskinetic patients exhibit abnormal motor regulation even in OFF condition, characterized by reduced damping and a tendency toward overshooting during visually guided tasks (Stevenson et al. 2011). These findings suggest that abnormal motor control mechanisms may persist independently of dopaminergic stimulation and may contribute to gait and balance disturbances even when dyskinesias are not clinically evident.

Previous functional MRI studies provide a neurobiological context for this hypothesis. In the OFF state, dyskinetic patients exhibit increased resting-state functional connectivity between the SNpc and putamen (Su et al. 2023), while task-based studies reveal abnormal activation of both the supplementary motor area (SMA) and prefrontal cortex, also specifically associated with LID severity (Su et al. 2023; Cerasa et al. 2012). In the ON state, dopaminergic stimulation may further modulate striato-cortical and motor networks, involving regions such as the putamen, SMA, and primary motor cortex (Chen Thomsen et al. 2024; Cerasa et al. 2015; Herz et al. 2014). These findings suggest that LID may be associated with broader motor-network alterations; however, the relationship between these functional changes and gait or balance impairment across medication states remains incompletely understood.

Taken altogether, these data suggest that LID reflects not only an abnormal motor output in ON state, but also a broader dysfunction of gait and balance control. To address this point, we assessed here the impact of LID on gait and postural parameters using wearable sensors in patients with advanced PD, examining them in both OFF and ON states. Moreover, we explored in a subgroup of patients representative for the whole sample, potential neurobiological substrates using structural and resting-state fMRI (rs-fMRI).

## Methods

Patients with PD were consecutively enrolled among those assessed for device-aided therapies due to disabling motor fluctuations or the presence of LID not adequately managed by oral medication (Deuschl et al. 2022). The study was conducted at the Movement Disorders Center, Turin University Hospital, between February 2021 and October 2024. The local institutional review board approved the study, and all participants gave written informed consent before entering the study. The study was conducted in accordance with the ethical standards laid down in the 1964 Declaration of Helsinki and its later amendments.

### Eligibility criteria

Inclusion criteria were: a diagnosis of idiopathic PD, according to the Movement Disorders Society (MDS) criteria (Postuma et al. 2015), and a Hoehn and Yahr (H&Y) score ≤ 4 in OFF state. Exclusion criteria were: the presence of neurological signs suggestive for a diagnosis other than idiopathic PD; orthopedic, rheumatologic, or other concomitant neurological conditions and/or previous surgical interventions significantly interfering with gait or balance assessments; a diagnosis of Parkinson’s disease dementia supported by a Montreal Cognitive Assessment (MoCA) score < 21 (Dalrymple-Alford et al. 2010); inability to walk independently (i.e., without the use of walking aid or assistance of another person) for 10 m or 60 s in OFF state; the presence of diphasic dyskinesia (emerging only during OFF-ON transitions or wearing-off, but absent at peak dose) (Vijayakumar and Jankovic 2016).

### Patient assessment

Demographic and clinical data included age, sex, disease duration, medical history, history of falls during the previous month, medication regimen and levodopa equivalent daily dose (LEDD) (Jost et al. 2023). PD severity and motor complications were assessed by the MDS-Unified Parkinson’s Disease Rating Scale (MDS-UPDRS) Parts I–IV. LID were evaluated by the Unified Dyskinesia Rating Scale (UDysRS) using both patient-reported history (part I) and direct clinical observation (part III and IV). The MoCA was used for global cognitive assessment (Dalrymple-Alford et al. 2010). Neurogenic orthostatic hypotension (nOH) was assessed using the Δheart rate/Δsystolic blood pressure ratio (ΔHR/ΔSBP), with nOH defined according to consensus criteria (systolic BP drop ≥ 20 mmHg or diastolic BP drop ≥ 10 mmHg within 3 min of standing, with ΔHR/ΔSBP < 0.5) (Imbalzano et al. 2024; Norcliffe-Kaufmann et al. 2018).

Clinical evaluations were performed in OFF (after ≥ 12 h withdrawal of levodopa, 24 h for other drugs) and ON medication state (after 1.5x morning dose of levodopa without adjunctive drugs).

Patients were categorized as dyskinetic (Dysk) or non-dyskinetic (No-Dysk) based on their UDysRS part I and/or part III/IV score > 0. The investigator performing clinical assessment was not involved in gait analysis or data analysis and statistics.

### Gait and balance analysis

Gait and balance were assessed through wearable inertial sensors (Opal, APDM’s Mobility Lab system, v2 2018; sampling rate of 128 Hz), placed bilaterally on feet and thighs, sternum, and lower back (Imbalzano et al. 2023, 2024; Virameteekul et al. 2025). Data were processed using manufacturer-provided software with algorithms validated for PD (Virameteekul et al. 2025; Morris et al. 2019). The following standardized tasks were administered:

- Sway test: patients were requested to stand upright on a firm surface, with their arms relaxed and eyes closed for 30 s. Root mean square (RMS) area (m/s^2^) was recorded to evaluate quiet stance balance.
- Timed-Up-and-Go (TUG) test: patients were required to stand up from a chair, walk 3 m, perform a tight 180° turn, walk back, and sit down again, all performed at a comfortable self-selected pace. In the present analysis, total TUG duration was used as a global measure of functional mobility and dynamic balance, incorporating postural transitions, straight walking, and turning.
- 2-minute walking test (2MWT): patients were instructed to walk at a comfortable self-selected pace back and forth along a 10-meter straight path for 2 min, performing tight 180° turns at each end, guided by floor markers. The test was conducted in a sufficiently large space to ensure continuous walking. Gait speed (m/s), double support (% gait cycle time), stride length (m), and step duration (s) were extracted from steady-state straight walking. Turning phases were automatically detected based on trunk angular displacement and angular velocity recorded by the sternum sensor and were excluded from the computation of steady-state spatiotemporal gait parameters. Mean values were averaged across all valid gait cycles. Gait variability was defined as the standard deviation of each gait parameter across the entire walking trial. Gait asymmetry was calculated as the absolute difference between right and left step parameters (i.e., largest minus smallest value).

### Exploratory structural and functional MRI

A subgroup of matched patients underwent exploratory structural and rs-fMRI at our Neuroradiology Unit, using a 3 T MRI scanner (Philips Achieva). Imaging was performed in the afternoon of the same day as gait assessment was run, under the patients’ usual dopaminergic regimen (ON state), to minimize discomfort associated with OFF state. Patients with severe tremor/dyskinesia, claustrophobia, or other MRI contraindications were excluded from MR scanning. Given the limited number of patients from the general cohort who received MRI, this analysis has to be regarded as exploratory.

The imaging component was designed to explore whether dyskinesia-related gait disturbances are associated with structural or functional alterations in motor control networks. Accordingly, analyses were restricted a priori to motor-related circuits, including the sensorimotor cortex, the basal ganglia, and cerebellum (Supplementary Fig. 1). MRI acquisition included a high-resolution T1-weighted volume, a 3D-fluid attenuated inversion recovery (3D-FLAIR) scan, and rs-fMRI (full acquisition parameters are reported in the Supplementary Methods). FLAIR scans were reviewed by an expert neuroradiologist to exclude any relevant co-pathology.

Voxel-based morphometry (VBM) was performed on T1-weighted volumes using CAT12/SPM12 to assess gray (GM) and white matter (WM) volumetric differences between Dysk and No-Dysk groups, controlling for total intracranial volume. Rs-fMRI data were preprocessed and analyzed using CONN toolbox (v22). After standard denoising, group-level independent component analysis (ICA) was used to identify resting-state functional networks, from which components corresponding to predefined motor-related networks were selected for between-group comparisons and correlation with quantitative gait metrics (Smitha et al. 2017).

### Statistical analysis

Continuous variables are reported as mean ± standard deviations (SD) and ranges, while categorical data are expressed as counts and percentages. Between-group comparisons (Dysk vs. No-Dysk groups) were performed using the Mann–Whitney U test for continuous data and Fisher’s exact test for categorical data.

Propensity score matching (PSM) with logistic regression was applied to reduce potential confounders and obtain clinically comparable groups (Garrido et al. 2014). A 1:1 nearest-neighbor matching without replacement was performed, with caliper of 0.1 SD of logit. Matching variables were selected based on their established relevance to gait impairment in PD: age, disease duration, motor severity (MDS-UPDRS part III total score in the OFF state), and the Postural Instability Gait Disorder (PIGD) score (sum of MDS-UPDRS items 3.9, 3.10, 3.12, and 3.13) (Gaßner et al. 2019; Dewey et al. 2014; Virameteekul et al. 2021). Sex was also included to account for the higher prevalence of LID among female patients (Tran et al. 2018). Matching quality was evaluated using standardized mean differences (SMD < 0.1) and by visual inspection of covariate distributions. Post-matching comparisons of gait and balance outcomes between Dysk and No-Dysk groups were performed using the Mann–Whitney U test. Within-group OFF vs. ON comparisons were analyzed using the Wilcoxon signed-rank test. Bonferroni correction was applied to control for multiple comparisons.

Neuroimaging analyses included voxel-wise comparisons of structural and functional MRI measures between groups, performed using two-sample t-tests. Outcomes of interest were gait/balance parameters significantly different between Dysk and No-Dysk groups in the ON state or showing significant differences across the OFF-ON transition (Δ values). For each voxel, a separate general linear model (GLM) was estimated with first-level connectivity or morphometry as dependent variables, and group or relevant clinical measures as predictors. Correlation analyses were also conducted to examine associations between MRI-derived metrics (gray matter volumes, rs-fMRI connectivity) and gait/balance outcomes. Inference was performed using multivariate parametric statistics with random-effects across subjects and sample covariance estimation across repeated measures. Cluster-level inferences relied on Gaussian Random Field theory (Soares et al. 2016; Nieto-Castanon 2020), with results thresholded using a combination of a cluster-forming *p* < 0.001 voxel-level threshold, and a familywise corrected p-FDR < 0.05 cluster-size threshold (Chumbley et al. 2010).

Statistical analyses were performed using IBM SPSS Statistics (version 30.0; IBM Corp., Armonk, NY). A two-tailed p-value < 0.05 was considered as statistically significant.

## Results

Of the 115 screened patients, 19 were excluded (17 not meeting the inclusion criteria, 2 with diphasic dystonic dyskinesia), thus leaving 96 participants (70 males, 26 females) for inclusion in the study. Seventy-four (77.1%) exhibited peak-dose dyskinesia (Dysk), while 22 were classified as No-Dysk (22.9%). Two patients who initially denied LID were reclassified as dyskinetic based on clinical observation during the ON phase of the levodopa challenge.

All 96 patients completed clinical and instrumented gait/balance assessment. At unadjusted comparison, the Dysk group had longer disease duration (12.7 ± 5.6 vs. 10.2 ± 3.0 years, *p* = 0.032), higher MDS-UPDRS part II score (16.0 ± 6.9 vs. 13.0 ± 6.3, *p* = 0.039), H&Y stage in both OFF (2.6 ± 0.7 vs. 1.8 ± 0.6, *p* = 0.002) and ON condition (2.1 ± 0.5 vs. 1.8 ± 0.6, *p* = 0.047), and MDS-UPDRS part IV (11.1 ± 2.6 vs. 8.1 ± 2.7, *p* < 0.001). Patients’ demographic and clinical characteristics are summarized in Table 1.

**Table 1:** Demographic and clinical characteristics in non-dyskinetic patients, all dyskinetic patients, and propensity-matched dyskinetic patients

	Dysk (all patients)	Dysk-matched (after PSM)	No-dysk	*P* value (dysk vs. no-dysk)	*P* value (dysk-matched vs. no-dysk)
*N* of patients (M/F)	74 (50/24)	22 (20/2)	22 (20/2)	/	/
Age (y)	60.5 ± 8.4	60.7 ± 7.7	60.8 ± 9.1	0.814	0.778
Disease duration (y)	12.7 ± 5.6	10.5 ± 3.6	10.2 ± 3.0	***0.032***	0.850
LEDD (mg)	1188 ± 408	1275 ± 457	1387 ± 485	0.358	0.398
MoCA (raw score)	24.1 ± 3.1	24.2 ± 3.2	24.5 ± 3.2	0.646	0.941
MDS-UPDRS I	13.1 ± 5.7	13.8 ± 5.7	13.3 ± 6.2	0.923	0.742
MDS-UPDRS II	16.0 ± 6.9	14.8 ± 7.6	13.0 ± 6.3	***0.039***	0.176
MDS-UPDRS III_OFF	47.6 ± 14.1	40.1 ± 10	41.9 ± 13	0.176	0.605
MDS-UPDRS III_ON	17.9 ± 9.8	14.3 ± 7.5	16.3 ± 9.3	0.364	0.698
Hoehn & Yahr stage – OFF	2.6 ± 0.7	1.9 ± 0.2	1.8 ± 0.6	***0.002***	0.697
Hoehn & Yahr stage – ON	2.1 ± 0.5	1.9 ± 0.6	1.8 ± 0.6	***0.047***	0.620
MDS-UPDRS IV	11.1 ± 2.6	11.5 ± 2.9	8.1 ± 2.7	***< 0.001***	***< 0.001***
PIGD score (OFF)	5.5 ± 2.9	5.1 ± 3.3	4.3 ± 2.5	0.072	0.568
PIGD score (ON)	2.7 ± 2.5	2.9 ± 2.4	2.1 ± 1.6	0.363	0.325
UDysRS I + II	13.1 ± 5.6	13.3 ± 6.3	/	/	/
UDysRS III + IV (ON)	9.2 ± 5.7	10.6 ± 7.0	/	/	/
N of fallers in the previous month (%)	21 (28.4)	7 (31.8)	5 (22.7)	/	/

Results are reported as mean ± standard deviation or absolute values, as appropriate. *P*-value < 0.05 was considered as statistically significant

*Dysk* Patients with dyskinesia, *F* Females, *LEDD* Levodopa equivalent daily dose, *M* Males, *MDS-UPDRS* Movement disorder society-unified parkinson’s disease rating scale, *MoCA* Montreal cognitive assessment, *No-Dysk* Patients without dyskinesia, *PIGD* Postural instability gait disorder (calculated as sum of MDS-UPDRS 3.9, 3.10, 3.12, 3.13), *PSM* Propensity score matching, *UDysRS* Unified dyskinesia rating scale

### Gait analysis

Before matching, the Dysk group showed significantly worse gait performance than the No-Dysk in both the OFF and ON state (Table 2), with lower gait speed, shorter stride length, longer TUG duration, and higher variability in stride length and step time.

**Table 2:** Instrumented gait and balance measures in non-dyskinetic patients and propensity-matched dyskinetic patients

	Dysk-matched (after PSM)	No-Dysk	*p* value (Dysk- matched vs. No-Dysk
Sway Test			
OFF - RMS Sway (m/s ^2^ )	1.03 ± 0.46	0.82 ± 0.37	0.159
ON - RMS Sway (m/s ^2^ )	1.63 ± 1.25	0.83 ± 0.21	***0.009***
3-meters Timed Up and Go			
OFF – TUG total duration (s)	24.03 ± 14.20	16.99 ± 6.59	***0.049***
ON – TUG total duration (s)	14.22 ± 4.89	11.24 ± 2.04	***0.009***
OFF – TUG Sit to stand duration (s)	1.10 ± 0.21	1.20 ± 0.27	0.105
ON – TUG Sit to stand duration (s)	1.07 ± 0.18	1.20 ± 0.21	***0.026***
2-Minute Walking Test			
OFF - Gait speed (m/s)	0.61 ± 0.28	0.84 ± 0.19	***0.007***
ON - Gait speed (m/s)	0.91 ± 0.28	1.02 ± 0.12	0.193
OFF - Speed asymmetry (m/s)	0.01 ± 0.01	0.02 ± 0.01	***0.030***
ON - Speed asymmetry (m/s)	0.02 ± 0.02	0.02 ± 0.01	0.525
OFF - Stride length (m)	0.70 ± 0.29	0.95 ± 0.21	***0.006***
ON - Stride length (m)	1.04 ± 0.24	1.18 ± 0.14	0.053
OFF - Stride asymmetry (m)	0.02 ± 0.01	0.03 ± 0.02	***0.025***
ON - Stride asymmetry (m)	0.03 ± 0.02	0.02 ± 0.02	0.492
OFF - Step time variability (s)	0.05 ± 0.04	0.03 ± 0.01	0.149
ON - Step time variability (s)	0.03 ± 0.02	0.02 ± 0.01	***0.021***
OFF - Double support (%GCT)	29.5 ± 9.4	25.0 ± 4.5	***0.047***
ON - Double support (%GCT)	25.5 ± 7.22	22.8 ± 3.60	0.511

Results are reported as mean ± standard deviation. *p*-value < 0.05 was considered as statistically significant

*Dysk* Patients with dyskinesia, *GCT* Gait cycle time, *No-dysk* Patients without dyskinesia, *PSM* Propensity score matching, *RMS* Root mean square, *TUG* Timed-up-and-go

After PSM, 22 patients from the Dysk group (Dysk-matched, 20 males, 2 females) were matched to 22 patients from the No-Dysk group, yielding well-balanced groups across baseline covariates (Table 1), except for MDS-UPDRS part IV, which by definition remained significantly higher in the Dysk-matched group (*p* < 0.001). Among Dysk-matched patients, 19 presented with choreiform movements, while 3 presented with a mixed phenomenology of choreiform and dystonic movements.

**Fig. 1:**
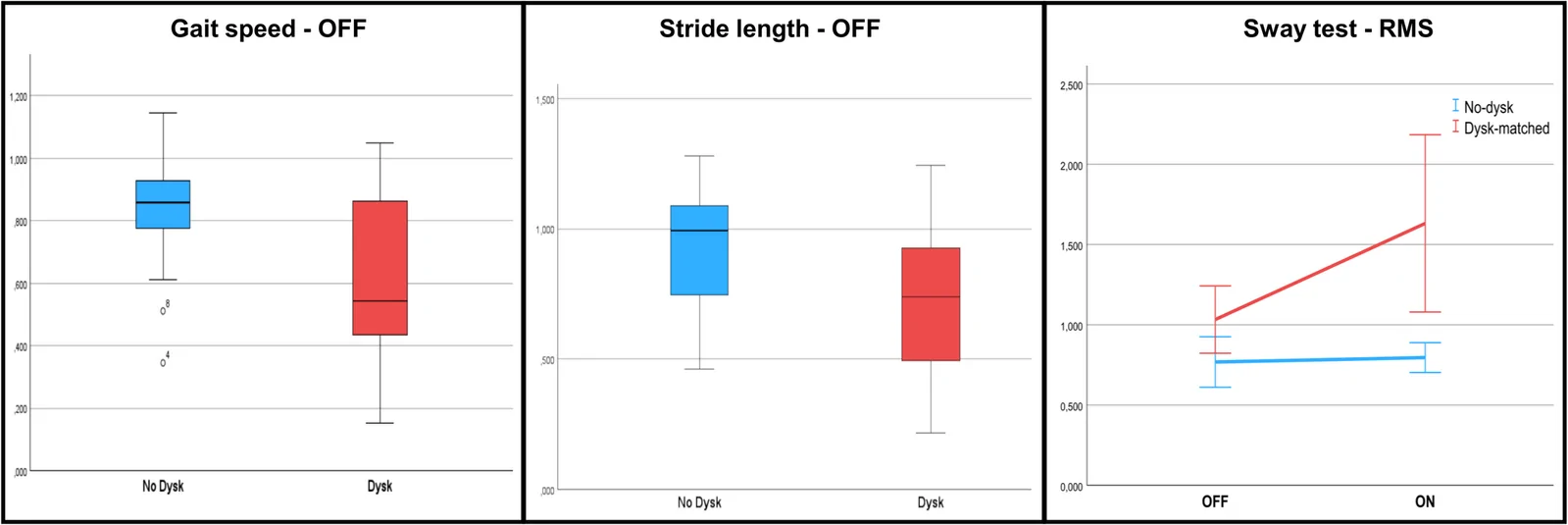
Gait differences in the OFF state and sway changes from OFF to ON in matched groups. Boxplots illustrating principal gait differences between non-dyskinetic patients (No-Dysk) and dyskinetic (Dysk) after propensity score matching (OFF state gait speed in m/s and stride length in m), and the change in Root mean square (RMS) sway (m/s^2^) from OFF to ON medication state (Dysk group in Red, No-Dysk group in blue)

In OFF state, Dysk-matched patients showed significantly lower gait speed (0.61 ± 0.28 vs. 0.84 ± 0.19 m/s, *p* = 0.007, *r* = 0.41), shorter stride length (0.70 ± 0.29 vs. 0.95 ± 0.21 m, *p* = 0.006, *r* = 0.42), longer double support time (29.5 ± 9.4 vs. 25.0 ± 4.5%GCT, *p* = 0.047, *r* = 0.30) and longer TUG duration (24.03 ± 14.20 vs. 16.99 ± 6.59 s, *p* = 0.049, *r* = 0.30) (Table 2; Fig. 1). Moreover, Dysk-matched patients exhibited lower gait speed asymmetry (0.01 ± 0.01 vs. 0.02 ± 0.01 m/s, *p* = 0.030, *r* = 0.33) and stride length asymmetry (0.02 ± 0.01 vs. 0.03 ± 0.02 m, *p* = 0.025, *r* = 0.34).

In ON state, the Dysk-matched group showed greater RMS sway (1.63 ± 1.25 vs. 0.83 ± 0.21 m/s^2^, *p* = 0.009, *r* = 0.39) and higher step time variability (0.03 ± 0.02 vs. 0.02 ± 0.01 s, *p* = 0.021, *r* = 0.35).

Within-group comparisons showed that both groups improved, when switching from OFF to ON state, in gait speed, stride length, double support time, and stride length variability (all *p* < 0.05). No significant OFF-ON changes were observed for step time or asymmetry metrics. RMS sway increased significantly only in the Dysk-matched group (from 1.03 ± 0.46 to 1.63 ± 1.25 m/s^2^, *p* = 0.030) (Fig. 1).

### Exploratory MRI analysis

Among the 44 patients matched for age, sex, disease duration, MDS-UPDRS part III OFF-total score and PIGD score, 30 (15 Dysk-matched, 15 No-Dysk) underwent MRI scanning. Three datasets were excluded due to poor T1 image quality, leaving 27 suitable scans for VBM analysis (13 Dysk-matched, 14 No-Dysk), which revealed no significant between group differences in regional GM or WM volumes.

For the rs-fMRI analysis, eight additional participants were excluded after automated EPI images quality check, resulting in a final sample of 19 patients (10 Dysk-matched and 9 No-Dysk). Patients included in this analysis did not differ from the overall matched cohort in key demographic and clinical characteristics, including age, disease duration, LEDD, and MDS-UPDRS part III in both OFF and ON states, reducing the likelihood of selection bias related to imaging quality exclusions.

**Fig. 2:**
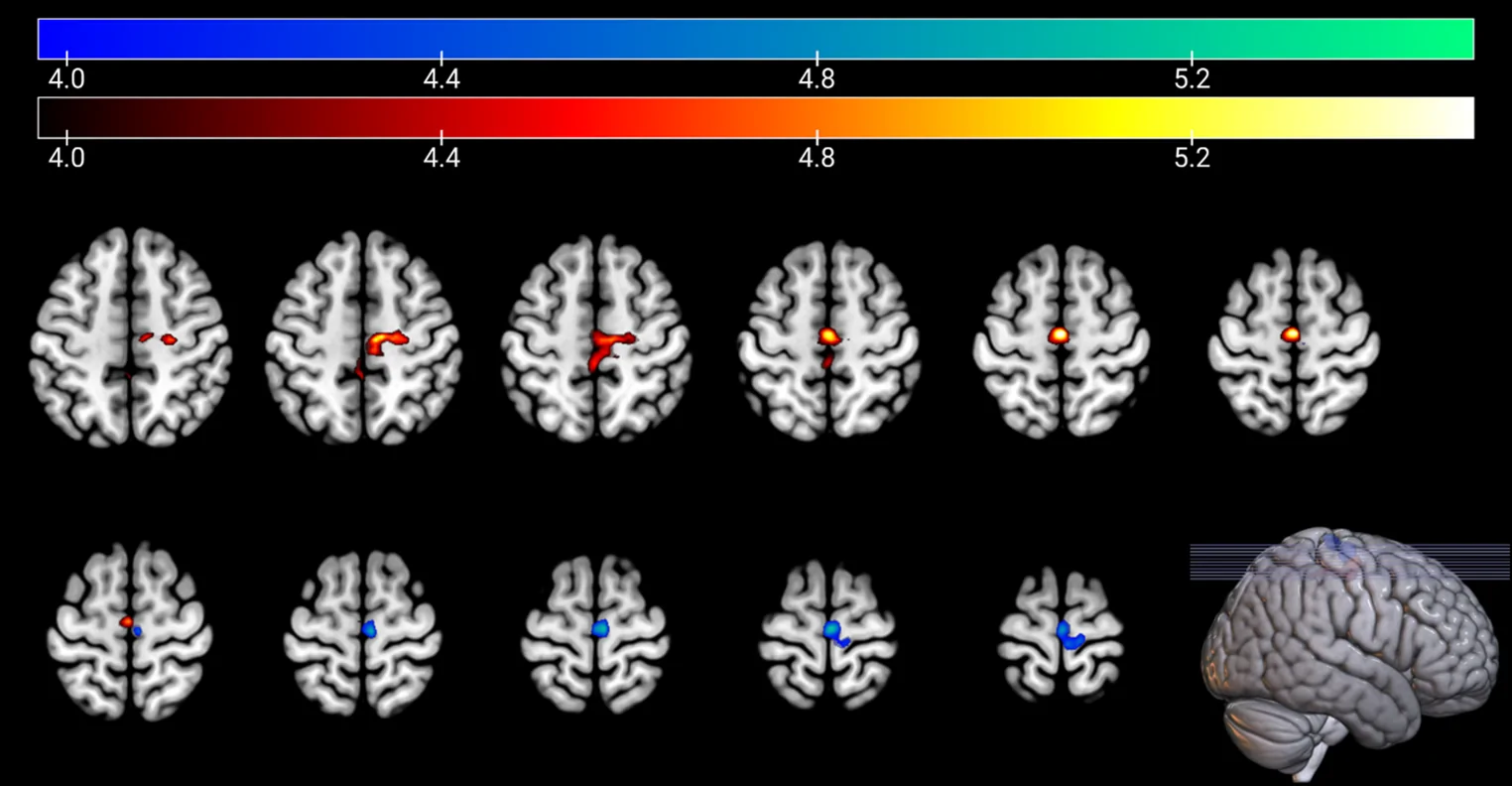
Resting-state fMRI differences between matched dyskinetic and non-dyskinetic patients. Resting-state functional MRI group differences between 10 dyskinetic (Dysk) and 9 non-dyskinetic (no-Dysk) patients. Neurological convention (left is left). Clusters with p-FDR value < 0.05 in motor and premotor area are displayed on the MNI template. Dysk < No-Dysk effects (cold, in blue) refer to the cerebellar lateral network, whereas Dysk > No-Dysk effects (hot, in red/yellow) refer to the sensory-motor medial network, showing significant differences in motor and premotor cortical regions

Between-group comparison revealed, in the central cortical portion of the sensory-motor networks (right paracentral lobule, BA 4), higher functional connectivity in Dysk-matched group compared to No-Dysk. Additionally, within the cerebellar network, the Dysk-matched group showed reduced functional connectivity in the left SMA (BA 6) (Supplementary Table 1, Fig. 2).

Correlation analyses corrected for multiple comparisons and restricted to the 19 patients with usable rs-fMRI data, returned significant findings within the cerebellar networks (see Supplementary Table 1). In ON condition, connectivity in the right cerebellum lobule IX correlated positively with double support.

Regarding transition effects (Δ OFF-ON), connectivity in the anterior cerebellar vermis correlated positively with increases in RMS sway, while connectivity in the right lateral cerebellum (lobule VIII) correlated negatively with sway increments.

## Discussion

In this study, we investigated the impact of LID on gait and balance in advanced PD, across both OFF and ON states, and explored potential neural correlates using structural and functional MRI. After minimization of baseline imbalances by PSM, patients with LID exhibited in OFF state slower gait speed (−27%), shorter stride length (−26%), longer double support (+ 4.5%GCT), and longer TUG compared with matched non dyskinetic patients. These differences suggest that patients who are prone to LID may present with intrinsic gait deficits independent of overt dyskinetic movements. Moreover, these findings are consistent with the hypothesis that patients prone to LID may exhibit broader dysfunction of motor control networks, although our imaging data do not allow direct verification of this mechanism (Stevenson et al. 2011; Chen Thomsen et al. 2024).

Interestingly, dyskinetic patients showed more symmetric gait features in OFF, particularly in gait speed and stride length. Asymmetry is a hallmark of early PD (Marin et al. 2015), but its relative reduction in dyskinetic patients may indicate a more bilateral involvement of motor circuit over disease progression. This interpretation is consistent with imaging findings, showing more bilateral SMA and M1 activation in LID (Cerasa et al. 2012; Herz et al. 2014, 2015), which potentially indicate reorganization toward a more symmetric but less adaptable gait control strategy.

In ON state, dyskinetic patients showed significantly a greater RMS sway and a higher step time variability, which are both markers of instability and fall risk (Bradley et al. 2025). This supports evidence indicating that dopaminergic stimulation improves pace-related metrics (speed, stride length) but may also worsen postural stability in patients with LID (Curtze et al. 2015; Bonnet et al. 2017; Aderonmu and Curtze 2024). While levodopa is generally associated with reduced gait variability in Parkinson’s disease (Bryant et al. 2011), the effect of dyskinesia on temporal gait regulation has been less extensively investigated. Although the between-group difference in step time variability was modest, dyskinetic patients continued to exhibit greater temporal variability despite the overall improvement induced by levodopa. This finding may indicate a residual alteration in temporal gait regulation that is not fully normalized by dopaminergic therapy. However, its clinical interpretation should remain cautious, as step time variability was assessed during steady-state straight-line walking, whereas turning-specific metrics were not analyzed and might have provided additional or partly different information on dynamic balance impairment in patients with LID.

The analysis of OFF-ON motor transitions further underscores this dual effect of dopaminergic stimulation in dyskinetic patients. In the current study, both groups improved in gait speed and stride length, thus reflecting dopaminergic benefit on propulsion (Armstrong and Okun 2020). However, in ON condition, postural sway increased in dyskinetic patients only, replicating previous findings (Curtze et al. 2015; Aderonmu and Curtze 2024), and reinforcing the hypothesis that LID is not only an epiphenomenon of dopaminergic excess, but reflects abnormal network dynamics and impairment of inhibitory control (Cenci et al. 2020; Herz et al. 2014).

In the exploratory neuroimaging analyses, VBM revealed no structural group differences, suggesting that gait and balance abnormalities in LID are unlikely to be explained by patterns of regional brain atrophy. Conversely, rs-fMRI analysis revealed functional connectivity changes within motor control networks, although our findings should be interpreted with caution given the modest sample size and the fact that all scans were acquired in ON state. Dysk-matched patients showed increased connectivity in the right paracentral lobule, corresponding to medial motor regions controlling foot and leg function, and decreased connectivity in SMA (Roux et al. 2020). The former finding may represent short-range compensatory synchronization of motor regions, while the latter finding is consistent with previous evidence implicating impaired SMA function in LID, which affects motor initiation and inhibitory control (Cenci et al. 2020; Cerasa et al. 2012; Turco et al. 2018).

Correlation analyses suggested possible associations between cerebellar network connectivity and gait/balance measures. Given that rs-fMRI data were acquired only in ON state, correlations with OFF-state gait measures are physiologically ambiguous and were not considered in the analysis. Connectivity in cerebellar lobule IX correlated with prolonged double support in ON state, suggesting a potential association between cerebellar network organization and automatic postural adjustments under dopaminergic stimulation (Takakusaki 2017). OFF-ON transition analyses provided additional exploratory information on dopaminergic responsiveness. Connectivity in the anterior cerebellar vermis was associated with larger sway increments after levodopa, consistent with impaired cerebellar modulation of postural stability (Maiti et al. 2021; Takakusaki 2017), while lateral cerebellar connectivity was associated to reduced sway increments, suggesting differential cerebellar contributions to postural responses after dopaminergic stimulation. Taken together, the gait/postural findings and the exploratory neuroimaging results are consistent with the possibility that LID is associated with network-level alterations involving the motor cortex, SMA, and cerebellum, in addition to striatal hypersensitivity to dopaminergic stimulation (Cenci et al. 2020; Herz et al. 2014). However, given that MRI was acquired only in the ON state, these observations should be considered hypothesis-generating rather than mechanistic evidence. In OFF state, reduced stride length and gait speed suggest intrinsic locomotor deficits, while in ON state, levodopa may improve propulsion and paradoxically destabilize postural control. This dual mechanism of propulsion gain and postural cost highlights the clinical complexity of LID, where dopaminergic therapy may simultaneously restore and destabilize motor control. Clinically, if confirmed in larger and longitudinal cohorts, these findings would support the need for tailored treatment strategies in advanced PD with LID.

Several limitations should be considered when interpreting these findings.

First, the relatively small sample size limited the statistical power and generalizability of results, in particular for neuroimaging data. This limitation may reduce representativeness of our subsample of patients despite the absence of systematic demographic or clinical differences with the whole cohort of subjects. MRI was acquired exclusively in ON state, limiting our ability to determine how network connectivity patterns differ between OFF and ON states, and whether the observed alterations are driven primarily by dopaminergic stimulation, chronic network reorganization, or their interaction. This limitation also precluded an interpretation of correlations with OFF-state gait and balance parameters, while the correlation with OFF-ON transition analyses should be interpreted only as a measure of dopaminergic responsiveness. Moreover, the absence of a healthy control group precludes interpretation of these findings relative to normative populations.

Second, although PSM minimized baseline demographic and clinical differences, residual confounding cannot be fully excluded, and the reduction in sample size resulting from matching limited the statistical power of subsequent analyses. Third, the cross-sectional design precludes causal inferences or assessment of disease progression. Longitudinal studies will be necessary to determine whether the identified gait and balance alterations are associated with future LID progression or clinically relevant mobility outcomes. Fourth, the assessments of gait and balance under standardized conditions may not fully capture the complexity of real-world mobility and fall dynamics. Fifth, dyskinesia was rated clinically but not quantified through objective kinematic measures, and different dyskinetic patterns (e.g., choreic versus dystonic) were not specifically analyzed, although dystonic features were present in a minority of patients, their potential differential impact on gait and balance cannot be completely excluded. Finally, it should be noted that our cohort consisted of patients evaluated for advanced device-aided therapies, who were able to walk independently in OFF state. This selection likely reduced the number of patients with more severe mobility impairments or frequent falls. Consequently, while our findings provide valuable insight into gait and postural control in dyskinetic patients within a referral context, they may not be fully generalizable to the broader Parkinson’s disease population.

Future research combining OFF-ON imaging in larger independent cohorts, along with concurrent biomechanical recordings, will help clarify the dynamic interplay between motor network reorganization, LID expression, and gait/postural control.

Despite these limitations, our findings provide novel evidence that gait and postural control in patients with LID are impaired not only in ON but also in OFF state, when dyskinesia is clinically absent. This supports the view that LID reflects broader motor network dysfunction beyond involuntary movements. Our exploratory imaging findings further suggest a potential association between SMA/cerebellar connectivity and these clinical manifestations. While these results require replication in larger and longitudinal studies, they underline the importance of therapeutic strategies that target both propulsion and stability across medication states.

## Supplementary Information

Below is the link to the electronic supplementary material.

Supplementary Material 1

## Data Availability

The data that support the findings of this study are available in anonymized dataset from the corresponding author, upon reasonable request.
