# Supplementary material for: Levodopa-induced dyskinesia is associated with worse gait and balance in Parkinson’s disease
Source: J Neural Transm (Vienna). 2026 Jun 30;133(9):2589–98. doi: 10.1007/s00702-026-03217-w (PMC13687194; doi:10.1007/s00702-026-03217-w)
Supplement: Supplementary file 1 [file 702_2026_3217_MOESM1_ESM.docx]

**Supplementary Material for Review**

**Supplementary Methods**

***Structural and functional MRI***

The MRI protocol included the following acquisitions: i) 3D-fluid attenuated inversion recovery (FLAIR) scan (TR = 5000 ms, TE = 355 ms, TI = 1700 ms, Matrix = 240 × 240 × 180, in–plane FOV = 240 × 240 mm^2^, slice thickness = 1.0 mm, ETL = 182, flip angle = 90°); ii) High-resolution T1-weighted volume (similar to MPRAGE, TR = 8.2 ms; TE = 3.8 ms; Matrix = 240 × 240 × 180, in–plane FOV = 240 × 240 mm^2^, slice thickness = 1.0 mm; ETL = 200, flip angle = 8°); iii) T2^*^ weighted echo planar imaging (EPI) volumes sensitized to BOLD contrast (TR = 2400 ms, TE = 31 ms, matrix = 80 × 80, voxel size = 3 × 3 mm^2^, 40 axial slices parallel to AC-PC line, slice thickness = 3.0 mm, gap 0.5 mm, flip angle = 80°).

After discarding the first 3 dummy scans, BOLD EPIs were collected during rest for an 8 min period, resulting in a total of 200 volumes. During this acquisition, subjects were instructed to keep their eyes closed, remain awake, and not focus on specific thoughts.

FLAIR images were carefully reviewed by an expert neuroradiologist to exclude the presence of remarkable co-pathology (e.g., cerebrovascular disease or other unexpected macroscopic abnormalities).

T1-weigthed volumes were pre-processed using the voxel-based morphometry (VBM) pipeline included in CAT12.9 (Computational Anatomy Toolbox 12, <https://neuro-jena.github.io/cat/>) (Gaser et al. 2024), a toolbox of SPM12 (<https://www.fil.ion.ucl.ac.uk/spm/software/spm12/>) (Penny et al. 2011). The pipeline includes automated tissue segmentation, spatial normalization to MNI space, and spatial smoothing using an 8 mm full width at half-maximum (FWHM) Gaussian kernel. Grey (GM) and white matter (WM) maps were used to assess between-group differences in regional volumetrics and associations with clinical and gait analysis parameters. In all VBM analyses, total intracranial volume (TIV = GM + WM + CSF) was included as a covariate of no interest. Considering the balanced groups after propensity matching, no other covariate was considered in imaging analyses (see statistical analysis section).

Resting-state fMRI (Rs-fMRI) data were analyzed using the CONN toolbox (release 22.v2407, <https://web.conn-toolbox.org/home>) (Whitfield-Gabrieli S and Nieto-Castanon 2012). Preprocessing included unwarping, motion correction, normalization to MNI space, scrubbing of outlier scans (using ART, 97^th^ percentile), resampling to 2 mm isotropic voxels, and spatial smoothing with an 8 mm FWHM Gaussian kernel (Alahmadi 2021). In addition, functional data were denoised using a standard denoising pipeline (Nieto-Castanon 2020), including the regression of white matter timeseries (5 CompCor noise components), CSF timeseries (5 CompCor noise components), motion parameters and their first order derivatives (12 factors), outlier scans (below 10 factors), session effects and their first order derivatives (2 factors), and linear trends (2 factors) within each functional run. Finally, BOLD timeseries were band-pass filtered between 0.008 Hz and 0.09 Hz.

Group-level independent component analyses (group-ICA or GICA) were performed to estimate 25 temporally coherent networks from the fMRI data combined across all subjects (Calhoun et al. 2021). A fast-ICA fixed-point algorithm () with hyperbolic tangent (G1) contrast function was used to identify spatially independent group-level networks (Hyvarinen 1999), and GICA3 back-projection was applied to compute ICA maps (or ICA components, IC) associated with these same networks separately for each individual subject (Erhardt et al. 2011). For the current study, we used an automated selection algorithm included in CONN which identifies the best spatial match between CONN’s network template and each group level spatial map, in order to detect all components related to sensory-motor networks. After visual confirmation, 6 ICs (Supplementary Figure 1) were retained for statistical analyses, including the cerebellum (3 ICs: anterior, posterior, lateral), the basal ganglia (1 IC), and sensory-motor areas (2 ICs: medial, lateral).

**Supplementary Tables**

**Supplementary table 1. SPM statistical results**

| **MRI measure** | **Analysis** | **Cluster (MNI coordinates x,y,z mm)** | **Cluster size (voxels)** | **Cluster p-FDR value** | **Peak AAL (BA) areas** |
| --- | --- | --- | --- | --- | --- |
| **ICA cerebellum (lateral)** | Dysk > No-Dysk | +00 -14 +58 | 295 | <0.001 | L SMA (6) |
|  | Corr OFF - Speed asymmetry | -06 -38 -16 | 228 | 0.003 | L cerebellum vermis III-IV |
|  | Corr OFF - RMS Sway | -42 +42 -10 | 413 | <0.001 | L frontal inferior orbital cortex (47) |
|  | Corr ON - Double support | +18 -46 -44 | 302 | 0.006 | R cerebellum lobule IX |
|  | Corr Delta - RMS Sway | -02 -36 -12 | 268 | 0.003 | L cerebellum vermis III |
|  | Acorr Delta - RMS Sway | +26 -44 -44 | 510 | <0.001 | R cerebellum lobule VIII |
| **ICA**  **sensori-motor (medial)** | Dysk < No-Dysk | +04 -28 +74 | 179 | 0.018 | R paracentral lobule (4) |

Acorr: negative correlation; AAL: Automated anatomical labelling; BA: Brodmann area; Corr: positive correlation; Delta: parameter change from OFF to the ON state; Dysk: Patients with dyskinesia; ICA: Independent component analysis, L = Left; No-dysk: Patients without dyskinesia; R: Right; RMS: Root mean square; SMA: Supplementary Motor Area.

**Supplementary Figures**

**Supplementary Figure 1. ICA components selected for rs-fMRI analysis**

Independent Component Analysis (ICA) components selected for resting state functional MRI (rs-fMRI) analysis after automatic matching with sensory-motor spatial template.

IC10 = cerebellar posterior network, IC3 = cerebellar anterior network, IC1 = cerebellar lateral network, IC20 = sensory-motor lateral network, IC22 = sensory-motor medial network, IC24 = basal ganglia network.

**References for supplementary methods**

**References:**

1. Gaser C, Dahnke R, Thompson PM, Kurth F, Luders E, Alzheimer’s Disease Neuroimaging Initiative (2024) CAT: a computational anatomy toolbox for the analysis of structural MRI data. GigaScience 13:giae049. https://doi.org/10.1093/gigascience/giae049.
2. Penny WD, Friston KJ, Ashburner JT, Kiebel SJ, Nichols TE, editors (2011) Statistical parametric mapping: the analysis of functional brain images. Elsevier.
3. Whitfield-Gabrieli S, Nieto-Castanon A (2012) Conn: A functional connectivity toolbox for correlated and anticorrelated brain networks. Brain Conn 2(3):125–141. https://doi.org/10.1089/brain.2012.0073.
4. Alahmadi AAS (2021) Effects of different smoothing on global and regional resting functional connectivity. Neuroradiology 63(1):99–109. https://doi.org/10.1007/s00234-020-02523-8.
5. Nieto-Castanon A (2020) Handbook of functional connectivity magnetic resonance imaging methods in CONN. Hilbert Press pp 17–25.
6. Calhoun VD, Adali T, Pearlson GD, Pekar JJ (2001) A method for making group inferences from functional MRI data using independent component analysis. Hum brain mapp 14(3):140–151. https://doi.org/10.1002/hbm.1048.
7. Hyvarinen A (1999) Fast and robust fixed-point algorithms for independent component analysis. IEEE Trans Neural Netw 10(3):626–634. https://doi.org/10.1109/72.761722.
8. Erhardt EB, Rachakonda S, Bedrick EJ, Allen EA, Adali T, Calhoun VD (2011) Comparison of multi‐subject ICA methods for analysis of fMRI data. Hum brain mapp 32(12):2075–2095. https://doi.org/10.1002/hbm.21170.
